# The Decisive Case-Control Study Elaborates the Null Association between *ADAMTS5* rs226794 and Osteoarthritis in Asians: A Case-Control Study and Meta-Analysis

**DOI:** 10.3390/genes12121916

**Published:** 2021-11-28

**Authors:** Chung-Cheng Kao, Hsiang-En Hsu, Yi-Chou Chen, Ming-Yu Tu, Su-Wen Chuang, Sui-Lung Su

**Affiliations:** 1 Tri-Service General Hospital Songshan Branch, National Defense Medical Center, Taipei 105309, Taiwan; kao8267kq@gmail.com; 2 School of Public Health, National Defense Medical Center, Taipei 114201, Taiwan; eve998877@gmail.com (H.-E.H.); suwen7@gmail.com (S.-W.C.); 3 Department of Orthopedics, Taoyuan General Hospital, Ministry of Health and Welfare, Taoyuan 325208, Taiwan; secretdovac@gmail.com; 4 Institute of Medical Sciences, National Defense Medical Center, Taipei 114201, Taiwan; 5 Department of Orthopedics, Kaohsiung Armed Forces General Hospital Gangshan Branch, Kaohsiung 820004, Taiwan; du0807@yahoo.com.tw; 6 Graduate Institute of Life Sciences, National Defense Medical Center, Taipei 114201, Taiwan

**Keywords:** osteoarthritis, ADAMTS5, gene polymorphism, case-control study, meta-analysis, trial sequential analysis

## Abstract

Background: Osteoarthritis is an important health issue for the elderly. Many studies indicate that genetics is an important risk factor for osteoarthritis, and a disintegrin and metalloproteinase with thrombospondin motifs 5 (*ADAMTS5*) is one gene that is most frequently implicated. Many recent studies have examined the relationship between a polymorphism in the *ADAMTS5* gene (rs226794) and the risk for developing osteoarthritis without definitive results. Objective: In this case-control study, we examined the correlation between the *ADAMTS5* gene polymorphism, rs226794, and knee osteoarthritis. We used a meta-analysis and trial sequential analysis to determine whether *ADAMTS5* rs226794 expression increases susceptibility to osteoarthritis. Methods: This study consisted of two parts: a case-control study and a meta-analysis. The case-control study included subjects who underwent knee radiography at the Health Examination Center of the Tri Service General Hospital from 2015 to 2019. The Kellgren–Lawrence (KL) grading system was used as diagnostic criteria. Patients with unsuccessful gene sequencing were excluded. There were 606 subjects in the knee osteoarthritis group (KL ≥ 2) and 564 in the control group (KL < 2). Gene sequencing was performed using iPLEX Gold to determine the association between the gene polymorphism of *ADAMTS5* rs226794 and knee osteoarthritis. For the meta-analysis, databases such as PubMed, Embase, and Cochrane were queried to identify studies that examined the relationship between *ADAMTS5* rs226794 and osteoarthritis. Next, the findings of the meta-analysis were incorporated with the results of the case-control study and samples from the published studies to estimate the association between the genetic polymorphism and osteoarthritis using an odds ratio and a 95% confidence interval. Results: We found a non-significant association between the G allele and knee OA (crude-OR: 0.93 (95% CI: 0.79–1.10) and adjusted-OR: 1.02 (95% CI: 0.76–1.36) in the allele model) in the present study, and the analysis of other genetic models revealed a similar trend. After including five published studies and our case-control study, the results with 2866 Asians indicated a conclusively null association between *ADAMTS5* rs226794 and knee OA) OR: 1.09 (95% CI: 0.93–1.26). The results for Caucasians also revealed a null association (OR: 1.21 (95% CI: 0.81–1.82)). Conclusions: This study indicates that the gene polymorphism, *ADAMTS5* rs226794, is not significantly associated with knee osteoarthritis. Additionally, assuming that the cumulative sample size in the allele model is sufficient, we confirmed that the G allele is not a risk factor for osteoarthritis. This study integrated all available evidence to arrive at this conclusion, and it suggests that no additional studies are necessary.

## 1. Introduction

Osteoarthritis (OA) is the most common joint disease occurring globally and is the primary cause of disability in the elderly [1]. Among all factors for OA, genetics are a particularly important factor, and the heritability of knee OA is approximately 45% [2]. Studies have suggested that OA is primarily influenced by genetic risk factors because of common polymorphisms in multiple genes in the population [3]. In the 2018 GWAS review of knee osteoarthritis, the susceptibility genes for knee osteoarthritis analyzed by GWAS were considered to be supported by sufficient genetic evidence [4]. There are only nine identified genes, which is likely insufficient to fully explain the heredity of knee osteoarthritis. Therefore, it is necessary to identify more candidate genes and evaluate their effects.

The *ADAMTS5* gene, one of the disintegrins and metalloproteinases with thrombospondin motifs (*ADAMTS*), is located on the long arm of chromosome 21 (21q.21). In mouse experiments, ADAMTS5 expression causes cartilage destruction in OA models [5]. In humans, ADAMTS4/5 is the main enzyme that leads to the degradation of proteoglycans in OA, because of an increase in the mRNA and protein expression in the cartilage of OA patients [6]. Rodriguez et al. found that ADAMTS5 is the main aggrecanase in mouse cartilage. They examined the genetic variation of the gene and found that rs226794 in *ADAMTS5* may be a risk factor for OA using bioinformatics methods. However, the study did not find any significant association between *ADAMTS5* rs226794 and OA. The results indicated that it is necessary to further explore the function of this aggrecanase in human OA to determine whether it has a similar role as that observed in mouse studies [7].

To date, five studies have examined the association between the rs226794 polymorphism and knee OA [7,8,9,10,11]; however, a satisfactory consensus has been reached, especially in Asians and Caucasians. All five studies showed no association between *ADAMTS5* rs226794 polymorphism and knee OA [7,8,9,10,11]. The trial sequential analysis (TSA) provided an opportunity to evaluate whether the most recent conclusions are supported by the current cumulative sample data [12]. However, the current sample size was only 1697 for Asians and 746 for Caucasians [7,8,9,10,11], and it is uncertain whether this is enough to obtain a decisive conclusion. The aim of this study was to conduct a case-control study to validate the association between *ADAMTS5* rs226794 polymorphism and knee OA in Taiwan. We also performed a meta-analysis to increase the amount of evidence and evaluate whether the latest conclusions are supported by the current cumulative sample data using trial sequential analysis (TSA).

## 2. Materials and Methods

### 2.1. Case-Control Study

#### 2.1.1. Ethical Issues

This study was approved by the Institutional Review Board (TSGH-2-102-05-028) of the Tri Service General Hospital (TSGH). Volunteers signed the consent form after the investigators had provided an explanation of the study.

#### 2.1.2. Subjects

A total of 1170 participants (606 case and 564 control) consisting of controls and patients ≥65 years of age were enrolled in this study. All received Taipei City senior medical check-ups between January 2015 and December 2019 at the TSGH, a teaching hospital at the National Defense Medical Center (Taipei, Taiwan). The check-up is a government-driven welfare program for individuals ≥65 years of age who have been registered as Taipei City residents for >1 year.

We examined patients’ information, while the participants underwent check-ups. All participants received study information, understood the process, and provided written consent upon enrollment. Participants were excluded if a sufficient blood sample could not be obtained. The exclusion criteria included patients who had no knee X-ray data and for whom gene sequencing was unsuccessful. A total of 25 samples could not be genotyped by our genotyping method, so there were only 1170 (97.9%) samples included in the final genetic analyses.

All participants underwent a radiographic examination of both knees with anterior–posterior and lateral views analyzed, as well as weight bearing and foot-map positioning. Knee radiographs were read and scored by a radiologist using the Kellgren–Lawrence (KL) grading system [13]. In the KL system, radiographs receive scores of 0–4 points. For patients with different KL grades in each knee, the more-advanced grade was used for the evaluation. We used a radiographic KL grade of ≥2 to define knee OA. According to the above classification, the study included 606 knee OA patients and 564 healthy controls.

#### 2.1.3. Genomic DNA Extraction and Genotyping

Approximately 10 mL of peripheral blood were intravenously extracted from participants by a physician or nurse. Genomic DNA was isolated using standard procedures including proteinase K (Invitrogen, Carlsbad, CA) digestion and phenol/chloroform extraction. The rs226794 SNP was genotyped using the Massarray iPLEX Gold SNP genotyping method. Genotyping was performed under blind conditions. To validate the results, at least 10% of the samples were randomly selected for repeated genotyping, and the concordance rate was 97.2%.

#### 2.1.4. Statistical Analysis

Continuous variables for the general demographic data were expressed as the mean and standard deviation using a Student’s *t* test. Differences in genotype and allele frequencies between knee OA patients and healthy controls were tested using a χ2 test. Odds ratios (ORs) and 95% confidence intervals (CIs) for the risk of knee OA were calculated using logistic regression. Calculation of the genetic polymorphism and knee OA risk was expressed using the allele type, the genotype, and dominant/recessive models. A *p*-value of <0.05 was considered statistically significant. R 3.4.4 software was used for the statistical analyses.

### 2.2. Meta-Analysis

#### 2.2.1. Search Methods and Criteria for Study Consideration

The PRISMA checklist and the Meta-analysis on Genetic Association Studies Checklist is described in Supplementary Table S1 [14]. This study used the general population as the target sample and explored the association between *ADAMTS5* rs226794 and OA risk. PubMed, EMBASE, and Cochrane were searched using "*ADAMTS5* rs226794,” "Osteoarthritis,” and their synonyms as queries for studies only in English up to 19 November 2020 (see Table S2 for details). In addition, our research team manually reviewed the studies included in meta-analyses performed in the past to avoid omission of important studies. The inclusion criteria for the studies were: (1) A case-control or cross-sectional study; (2) a study using the KL grading system as its diagnostic criteria, such as case (KL ≥ 2) and control (KL < 2) groups; and (3) a study with adult subjects over 18 years of age.

#### 2.2.2. Data Extraction

This study had two reviewers who worked independently to collect data. The data collected from the literature included the first author’s surname, the year of publication, the country, the ethnicity of the research group, and the gene distribution of the case and control groups. All extracted studies were assessed using the Newcastle–Ottawa Scale, and all received scores of >6 points.

#### 2.2.3. Statistical Analysis

Each included article was described with an appropriate ratio or average value. The meta-analysis in this study used ORs with 95% CIs to examine the correlation between *ADAMTS5* rs226794 and OA. The I2 test was used to evaluate the heterogeneity, where I2 > 50% indicated that there was moderate to high heterogeneity. This study also used Egger’s regression and a funnel plot to examine the symmetry after the incorporation of the two parts. In addition, genetic models including allele, dominant, and recessive models were used to calculate the risk level of *ADAMTS5* rs226794 and OA by combining the calculation results by a random-effects model. In this study, the significance level was set at 0.05, and the statistical software used were the packages “metafor” [15] and “meta” [16] of R software version 3.3.1. (Vienna, Austria). This study used TSA to verify that the results obtained from the meta-analysis were a definite conclusion [17]. TSA was used for stratification analysis based on race (Caucasian and Asian). The type 1 error was set at 0.05. The power was set at 0.8. Heterogeneity in the Caucasian and Asian populations was set at 28% and 0%, respectively. A review of the past literature showed that the OR of the correlation between *ADAMTS5* rs226794 and OA was around 1.2. In the present study, considering that the G allele was a possible risk factor, the OR value was set at 1.2. The Taiwan Biobank database and the 1000 Genome database were used as references for the minor allele frequency, which is 0.30 for Asians and 0.11 for Caucasians.

## 3. Results

### 3.1. Case-Control Study

Table 1 shows the distribution of basic demographic variables of the case-control study population. This study enrolled a total of 1,170 subjects, of which 564 were in the control group with a mean age of 71.61 ± 6.79 years (277 men and 287 women). The remaining 606 were in the case group with a mean age of 73.45 ± 7.30 years (219 men and 387 women). The proportion of men in the case group was lower compared with that in the control group (*p* < 0.001), and the average age of the case group was higher compared with that in the control group (*p* < 0.001). Table 2 shows the association between *ADAMTS5* rs226794 and knee OA in the case and control groups. The distribution of the G allele in the control group and the case group was not significantly different (*p* = 0.890), and there was no significant correlation with OA (OR: 1.02 (95% CI: 0.76–1.36)). To further verify the results, a dominant model and a recessive model were used in which no significant difference was found after correcting for covariates. Therefore, our study found that *ADAMTS5* rs226794 was not significantly correlated with OA. To increase the level of evidence for the meta-analysis, the samples in the case-control study were added to the meta-analysis. TSA was then performed to identify whether or not the same can be concluded for the Asian population.

### 3.2. Meta-Analysis

**Figure 1:**
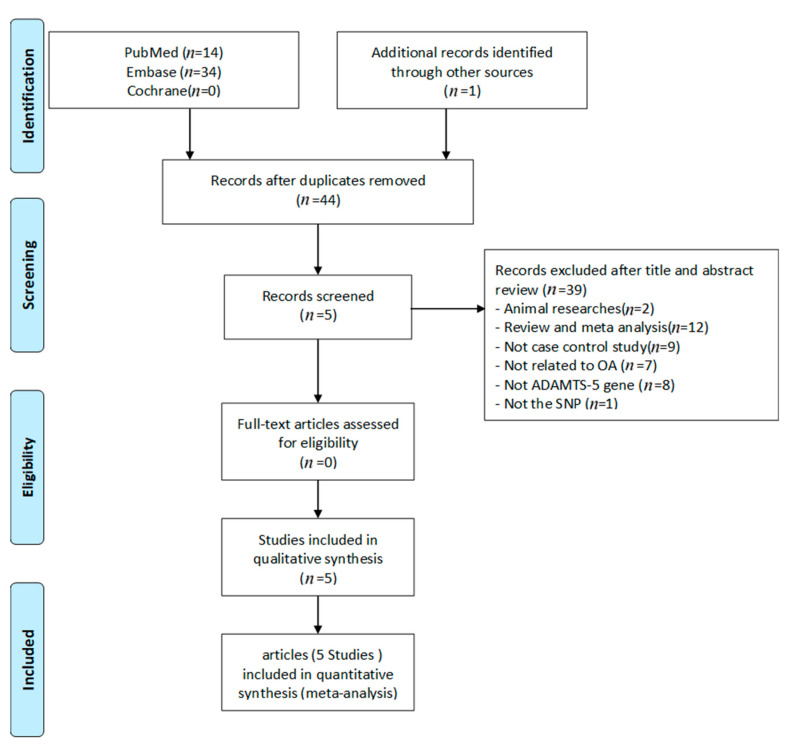
Flow diagram of the identification process for eligible studies.

**Figure 2:**
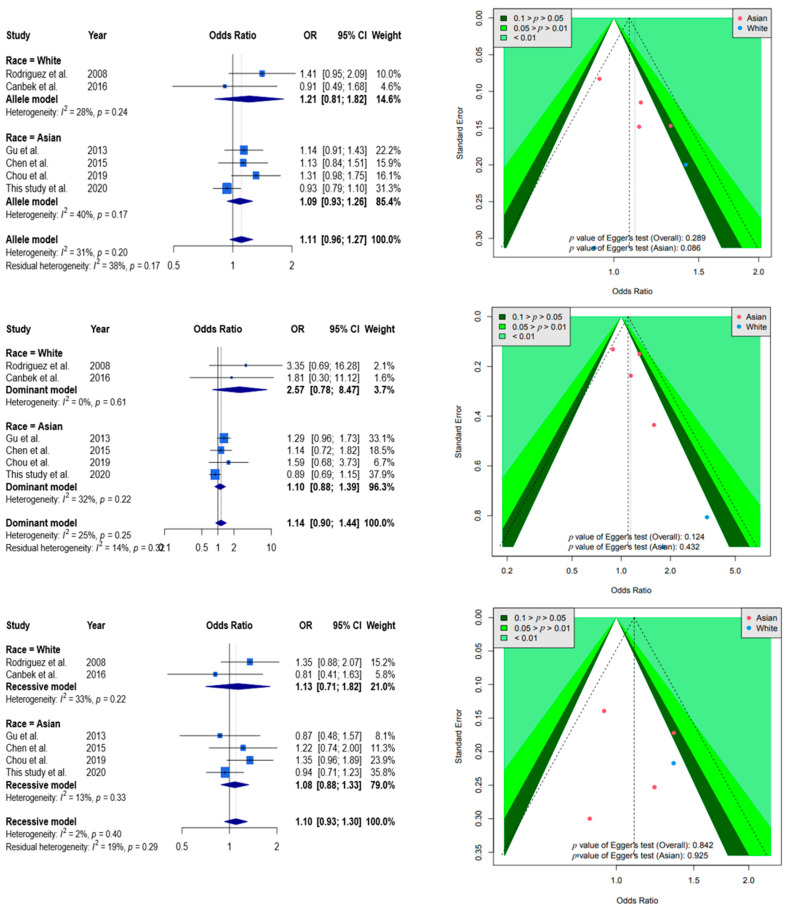
Forest plot and funnel plot showing the association between *ADAMTS5* rs226794 and OA.

The flow chart for the literature search of this study is shown in Figure 1. In the meta-analysis, 14 articles were initially collected from PubMed. Then, another 35 articles that were previously missed were collected by a manual review of Embase and the other meta-analyses, for a total of 49 articles. After the duplicate articles were excluded, a total of 44 articles were used for inclusion screening, which was carried out based on the titles and abstracts. Among the 44, 12 were excluded as they were review or meta-analysis articles, and another 25 were considered irrelevant, and the other 2 consisted of animal studies. Finally, five articles were included for analysis. The basic description of the articles included in the meta-analysis is shown in Table S3. The quality of the evaluation is shown in Table S4. Figure 2 shows the results of the meta-analysis. According to the allele model (allele G to allele A), with the six samples all incorporated, the results were not significant, OR = 1.11 (95%CI: 0.96–1.27). In terms of ethnic stratification, there were four samples of Asians, and the result was not significant, OR = 1.09 (95%CI: 0.93–1.26). There were two samples of Caucasians, and the result was not significant, OR = 1.21 (95%CI: 0.81–1.82).

A funnel plot was used to demonstrate the association between ORs and standard error in the allele model, with each point representing a study. No significant asymmetry was discovered between the articles. It is worth mentioning that only two Caucasian studies could not be tested by Egger’s regression. The other analyses based on a dominant and recessive model showed similar results as the allele model.

Selected results from the meta-analysis of *ADAMTS5* rs226794 and knee OA. The top left subplot is a forest plot based on an allele model assumption (reference: G allele), and the top right subplot is a funnel plot based on the allele model assumption. The results obtained with the dominant (AA +AG vs. GG) and recessive (AA vs. AG+ GG) models are presented in the middle and bottom. All results were insignificant.

### 3.3. TSA Evaluation

**Figure 3:**
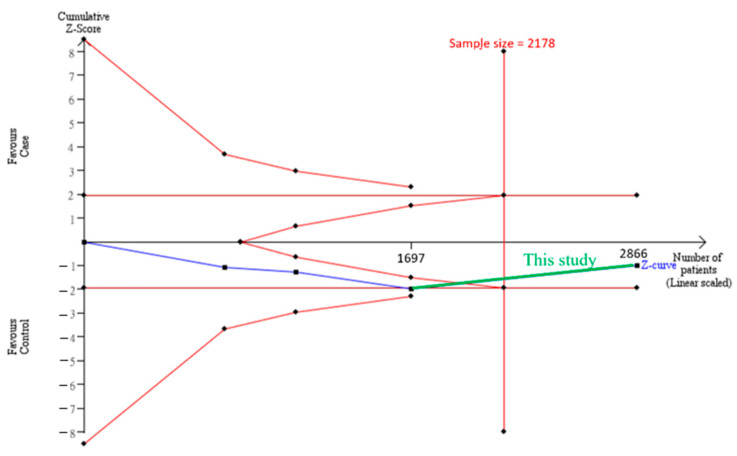
Trial sequential analysis (TSA) in Asians. We performed a TSA using an allele model assumption but replaced the allele count with the sample size (divided by 2). Detailed settings: significance level = 0.05; power = 0.8; ratio of controls to cases = 1; hypothetical proportion of controls with G allele = 0.30; least extreme OR to be detected = 1.2; I^2^ (heterogeneity) = 0%.

**Figure 4:**
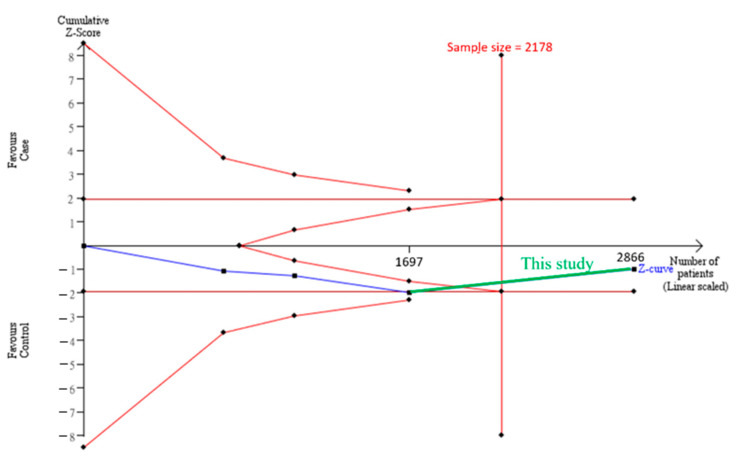
Trial sequential analysis (TSA) in Caucasians. We performed a TSA using an allele model assumption but replaced the allele count with the sample size (divided by 2). Detailed settings: significance level = 0.05; power = 0.8; ratio of controls to cases = 1; hypothetical proportion of controls with G allele = 0.19; least extreme OR to be detected = 1.2; I^2^ (heterogeneity) = 28%.

The cumulative sample size for the Asian population was only 1,697 before adding our case-control study samples, which could not establish a decisive conclusion. Our case-control study provided 1170 samples to enhance the information, and a total of 2867 allowed the Z curve to exceed the sample size required (Figure 3). This result shows that *ADAMTS5* rs226794 and knee OA is not significantly associated with Asians, and a decisive conclusion was confirmed. Our case-control study was the critical information for this meta-analysis. Figure 4 shows the result of TSA in Caucasians. The cumulative sample size did not reach the sample size required. This meta-analysis resulted in consistent insignificant results for all analyses.

## 4. Discussion

The results of this study show that the *ADAMTS5* rs226794 gene polymorphism is not significantly associated with OA, which is consistent with the results of previous studies. Compared with the previous studies published for Caucasians, we found that the gene polymorphism of *ADAMTS5* rs226794 was not significantly associated with OA, which was similar to the results of Rodriguez et al. and Canbek et al. [7,8]. Compared with the study of the Asian ethnic groups, the results of Gu et al., Chen et al., and Chou et al. indicated that the gene polymorphism of *ADAMTS5* rs226794 was not significantly associated with OA [9,11,18], which was consistent with the results of the present study. The meta-analysis in this study found that *ADAMTS5* rs226794 is not associated with OA, which is consistent with the results of the meta-analysis by Huo et al. showing that the gene polymorphism of *ADAMTS5* rs226794 is not significantly associated with OA [19].

In mouse experiments, ADAMTS5 expression leads to cartilage destruction in OA models [5], which indicates that ADAMTS5 may be involved in cartilage degradation. Additionally, animals with an insufficient expression of ADAMTS5 exhibited resistance to cartilage degeneration [20]. Because of the increased mRNA expression of ADAMTS5 in OA cartilage, ADAMTS5 also appears to be the main enzyme involved in the degradation of proteoglycans in human OA [5]. Rodriguez et al., considering that ADAMTS5 is the main aggrecanase for cartilage destruction in mouse experiments, explored the genetic variation of *ADAMTS5* and used bioinformatics methods to successfully identify a single nucleotide polymorphism (SNP) in *ADAMTS5* that may promote OA, in addition to discovering rs226794 in exon 7. However, the study found that there was no significant association between *ADAMTS5* rs226794 and OA [7]. It is necessary to further explore the function of this aggrecanase in human cartilage to clarify whether or not it plays the same role as that in the mouse model. The inflammatory factor, IL-1β, can inhibit the expression of the transcription factor, SOX9, thereby regulating the expression of miR-140. miR-140 inhibits the expression of ADAMTS5, leading to cartilage degradation. Several case-control studies have shown that ADAMTS5 is not significantly associated with knee OA, which may result from the increase in miR-140 expression caused by the Sox9 transcription factor, which, in turn, inhibits ADAMTS5 from affecting articular cartilage.

Our study has three strengths: (1) Estimation of the sample size by TSA: In the past, no meta-analysis has employed a method to estimate whether its sample size reached the benchmark for a conclusion. This study, after TSA evaluation, indicated that the sample sizes were insufficient in both studies of Caucasians and Asians. (2) In general, epidemiological studies only use their own samples for analysis, whereas meta-analysis studies only analyze published articles. This study combined the methods used in traditional observational studies and meta-analysis techniques to further increase the sample size to improve the level of evidence. Next, TSA was used to examine the sample size and confirmed that there was no significant association between the genetic polymorphism of *ADAMTS5* rs226794 and knee OA in the Asian population. (3) The results of the two parts were incorporated by using a random-effects model, which can avoid serious errors that may be caused by model selection based on the level of heterogeneity [21].

This study has two limitations. First, only English articles were included, thus articles published in other languages were not included in the meta-analysis, which may cause bias in the results obtained after incorporation. Second, the high heterogeneity could not be explained, which implies potential gene–gene and gene–environment interactions. Our previous study developed a revised version of meta-regression, known as case-weighted meta-regression, to analyze the gene–gene and gene–environment interactions using average population information [22]. We suggest that further studies should provide complete population characteristics for future meta-analyses.

## 5. Conclusions

The definitive null relationship between ADAMTS5 rs226794 and knee OA was validated in this study. The information regarding Caucasian studies was insufficient before this study; however, our case–control study provided the critical evidence to elaborate on this phenomenon in Asians. Our study integrated all of the current evidence to confirm this null relationship between ADAMTS5 rs226794 and knee OA and suggests that there is a need for future studies.

## Figures and Tables

**Table 1:** Distribution of the basic demographic variables of the study subjects.

	Knee OA Group (*n* = 606)	Control Group (*n* = 564)	*p*-Value
Gender (%)			<0.001 *
Male	219 (36.1%)	277 (49.1%)	
Female	387 (63.9%)	287 (50.9%)	
Age (mean±SD)	73.45 ± 7.30	71.61 ± 6.79	<0.001 *
BMI	24.58 ± 3.63	24.10 ± 3.31	0.021 *
SBP (mmHg)	133.39 ± 17.84	130.90 ± 16.96	0.016 *
DBP (mmHg)	77.13 ± 10.90	77.22 ± 11.09	0.882
KL Grade (%)			<0.001 *
0	0	23 (4.1%)	
1	0	541 (95.9%)	
2	452 (74.6%)	0	
3	83 (13.7%)	0	
4	71 (11.7%)	0	

Knee OA group: KL ≥ 2; control group: KL < 2; BMI, body mass index; SBP, systolic blood pressure; DBP, diastolic blood pressure. * *p*- value < 0.05.

**Table 2:** Association between genetic polymorphism of *ADAMTS5* rs226794 and knee osteoarthritis.

	Knee OA Group (*n* = 606)	Control Group (*n* = 564)	Crude-OR (95% CI)	*p*-Value	Adj-OR ^a^ (95% CI)	*p*-Value
Genotype				0.667		0.785
AA	173 (28.5%)	148 (26.2%)	1.00		1.00	
AG	298 (49.2%)	284 (50.4%)	0.90 (0.68–1.18)	0.438	0.91 (0.68–1.20)	0.496
GG	135(22.3%)	132 (23.4%)	0.87 (0.63–1.21)	0.421	0.96 (0.68–1.35)	0.805
Allele Model				0.406		0.890
A	644 (53.1%)	580 (51.4%)	1.00		1.00	
G	568 (46.9%)	548 (48.6%)	0.93 (0.79–1.10)	0.406	1.02 (0.76–1.36)	0.890
Dominant Model				0.377		0.551
AA	173 (28.5%)	148 (26.2%)	1.00		1.00	
AG+ GG	433 (71.5%)	416 (73.8%)	0.89 (0.69–1.15)	0.377	0.92 (0.70–1.21)	0.551
Recessive Model				0.646		0.890
AA+AG	471 (77.7%)	432 (76.6%)	1.00		1.00	
GG	135 (22.3%)	132 (23.4%)	0.94 (0.71–1.23)	0.646	1.02 (0.76–1.36)	0.890

Knee OA group KL ≥ 2; control group: KL< 2; ^a^: corrected age, gender, BMI; MAF (Taiwan Biobank): 47%; MAF (1000 Genome): 47%.

## Data Availability

Not available.

## Supplementary Materials

The following are available online at https://www.mdpi.com/article/10.3390/genes12121916/s1, Table S1. PRISMA 2009 Checklist. Table S2. Search strategies and detailed records. Table S3. Basic description of articles included in the meta-analysis. Table S4. Quality evaluation of articles included in the meta-analysis.
